# Thiol‐Amine‐Based Solution Processing of Cu_2_S Thin Films for Photoelectrochemical Water Splitting

**DOI:** 10.1002/cssc.202101347

**Published:** 2021-08-26

**Authors:** Xi Zhang, Wooseok Yang, Wenzhe Niu, Pardis Adams, Sebastian Siol, Zhenbin Wang, S. David Tilley

**Affiliations:** ^1^ Department of Chemistry University of Zurich Winterthurerstrasse 190 8057 Zurich Switzerland; ^2^ Surface Science and Coating Technologies Empa Swiss Federal Laboratories for Materials Science and Technology Überlandstrasse 129 8600 Dübendorf Switzerland

**Keywords:** copper sulfide, molecular inks, solution processing, photoelectrochemical, water splitting

## Abstract

Cu_2_S is a promising solar energy conversion material owing to its good optical properties, elemental earth abundance, and low cost. However, simple and cheap methods to prepare phase‐pure and photo‐active Cu_2_S thin films are lacking. This study concerns the development of a cost‐effective and high‐throughput method that consists of dissolving high‐purity commercial Cu_2_S powder in a thiol‐amine solvent mixture followed by spin coating and low‐temperature annealing to obtain phase‐pure crystalline low chalcocite Cu_2_S thin films. After coupling with a CdS buffer layer, a TiO_2_ protective layer and a RuO_*x*_ hydrogen evolution catalyst, the champion Cu_2_S photocathode gives a photocurrent density of 2.5 mA cm^−2^ at −0.3 V vs. reversible hydrogen electrode (V_RHE_), an onset potential of 0.42 V_RHE_, and high stability over 12 h in pH 7 buffer solution under AM1.5 G simulated sunlight illumination (100 mW cm^−2^). This is the first thiol‐amine‐based ink deposition strategy to prepare phase‐pure Cu_2_S thin films achieving decent photoelectrochemical performance, which will facilitate its future scalable application for solar‐driven hydrogen fuel production.

## Graphical abstract

**Phase‐pure and photoactive** Cu_2_S thin films are prepared by dissolving Cu_2_S powder in a thiol‐amine solvent mixture followed by spin‐coating and low‐temperature annealing. The champion photocathode delivers a photocurrent density of 2.5 mA cm^−2^ at −0.3 V vs. V_RHE_, an onset potential of 0.42 V_RHE_, and high stability over 12 h in a pH 7 buffer solution under AM1.5 G simulated sunlight illumination (100 mW cm^−2^).

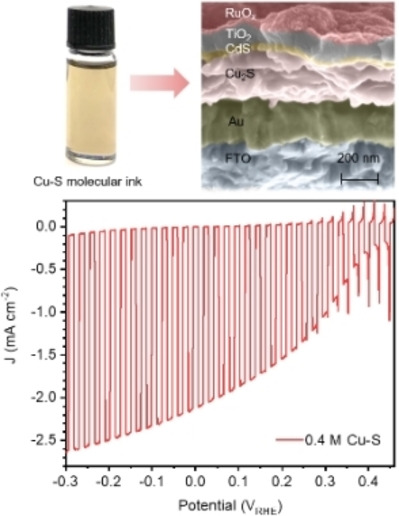

## Introduction

Although the solar power striking the Earth's surface is vastly superior to the global energy demand, the nature of solar energy, including variability, diurnal/seasonal intermittency, and low density, requires the conversion into storable vectors on a large scale for its effective utilization. Photoelectrochemical (PEC) water splitting is one promising strategy to convert the enormous amount of solar energy into storable hydrogen fuel. To implement a cost‐effective, large‐scale solar hydrogen fuel production infrastructure, earth‐abundant light absorbers and cheap, scalable fabrication methods are of crucial importance.

Copper(I) sulfide (Cu_2_S) is one promising p‐type light absorber for solar energy conversion. Consisting of only Cu and S, which are abundant and nontoxic,^[1]^ Cu_2_S is far superior to other potential chalcogenide light absorbers such as SnS, Sb_2_S_3_, Sb_2_Se_3_ and CuInGaSe_2_ (CIGS) with regards to the elemental earth abundance and cost.[^1^, ^2^] Numerous studies have shown that Cu_2_S has an indirect band gap of around 1.2 eV, a direct band gap of around 1.8 eV, and a high absorption coefficient (α) over 10^4^ cm^−1^,[^3^, ^4^] which indicate that it could theoretically achieve a maximum solar‐to‐electrical power efficiency of 30 %.^[5]^ When compared with another promising Cu‐based binary light absorber material, Cu_2_O (band gap≈2.0 eV),[^6^, ^7^] the smaller band gap of Cu_2_S is potentially beneficial for harvesting a larger portion of the solar spectrum. Indeed, Cu_2_S is an old hero in the field of solar energy conversion, since the first Cu_2_S/CdS heterojunction solar cell was reported in 1954.^[8]^ The efficiencies of Cu_2_S based solar cells reached over 10 % in the late 1970s and early 1980s,[^9^, ^10^, ^11^] which was comparable to the best Si‐based solar cells at that time. However, there are several nonstoichiometric copper sulfide phases such as djurleite Cu_1.96_S, digenite Cu_1.8_S, anilite Cu_7_S_4_ and covellite CuS which can also be formed.[^12^, ^13^] The increasing copper deficiency causes higher majority carrier concentration, and the higher density of shallow acceptors act as recombination centers.[^14^, ^15^, ^16^] Thus, the primary challenge to obtain efficient Cu_2_S thin film‐based solar energy conversion devices is to develop preparation methods for phase‐pure Cu_2_S thin films over large areas.

Many methods have been used to prepare Cu_2_S thin films, including cation exchange reaction,^[9]^ electrodeposition,^[17]^ spray pyrolysis deposition,^[18]^ pulsed chemical vapor deposition,^[19]^ physical vapor deposition,^[20]^ and atomic layer deposition (ALD).[^21^, ^22^] However, there are only few solar energy conversion devices based on the aforementioned methods, presumably due to the lack of phase purity. Thus, a simple fabrication process suitable for large‐area preparation of high‐purity Cu_2_S thin films should be developed for its practical applications.

Solution‐based film deposition is regarded as cost‐effective and high‐throughput. A particularly effective approach for chalcogenide thin films is the thiol‐amine‐based molecular ink method offering an opportunity to obtain phase‐pure high‐quality crystalline chalcogenide thin films.[^23^, ^24^, ^25^, ^26^, ^27^] It has been reported that more than 65 bulk inorganic materials including CdS, In_2_S_3_ and ZnS (insoluble in hydrazine) can be dissolved in the binary thiol‐amine solvent mixture to form molecular inks.^[28]^ Upon spin‐coating of these inks onto a variety of substrates followed by a low‐temperature annealing procedure, the solid‐state crystalline phase can be recovered. This straightforward dissolution and recovery strategy has been successfully applied to prepare various phase‐pure high‐quality crystalline chalcogenide thin films, such as Sb_2_Se_3_,[^23^, ^29^, ^30^, ^31^, ^32^] SnS,[^25^, ^33^] and CIGS,[^27^, ^34^] demonstrating the versatility of this cost‐effective and low‐energy consuming process.

In this work, phase‐pure stoichiometric crystalline Cu_2_S thin films were prepared from high‐purity Cu_2_S powder by using the thiol‐amine molecular ink method. Liquid‐phase Raman, thermogravimetric analysis (TGA) and differential thermal analysis (DTA) were used to study the properties of the Cu‐S molecular inks. X‐ray diffraction (XRD) demonstrated that the phase of as‐prepared Cu_2_S thin films is low chalcocite Cu_2_S, without Cu_1.96_S, Cu_1.8_S, CuS or any other impurity phases. It has also been confirmed by X‐ray photoelectron spectroscopy (XPS) that the oxidation state of copper in the Cu_2_S thin films is Cu^+^ and no copper oxide signals were observed. On top of the obtained Cu_2_S thin films, 40 nm of CdS as the buffer layer, 100 nm of TiO_2_ as the corrosion protection layer and RuO_*x*_ hydrogen evolution catalyst were deposited to construct Cu_2_S thin film‐based photocathodes. The champion photocathode revealed a photocurrent density of 2.5 mA cm^−2^ at −0.3 V *vs*. reversible hydrogen electrode (V_RHE_) and an onset potential of 0.42 V_RHE_ with extended stability over 12 h. To our knowledge, this is the first ink‐based deposition strategy for phase‐pure Cu_2_S thin films achieving decent PEC performance by thiol‐amine‐based solution processing.

## Results and Discussion

### Synthesis and characterization of the Cu‐S molecular ink

**Figure 1:**
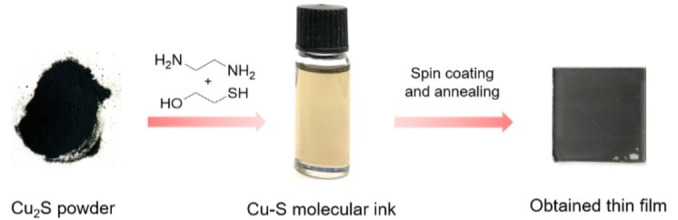
Images of Cu_2_S powder, Cu‐S molecular ink and the obtained low chalcocite Cu_2_S thin film on a 2.5×2.5 cm^2^ FTO substrate. The molecular ink preparation and the thin film preparation were performed in a N_2_‐filled glovebox.

The Cu‐S molecular ink was prepared by dissolving bulk Cu_2_S powder into a binary mixture of 2‐mercaptoethanol (Merc) and ethylenediamine (En) with a 1 : 4 volume ratio (1 : 4.2 molar ratio) followed by stirring overnight at room temperature in a N_2_‐filled glovebox. As shown in Figure 1, the Cu‐S molecular ink exhibits a transparent brownish color without sedimentation, indicating that the Cu_2_S powder has been fully dissociated into molecular species. Upon spin‐coating and annealing of the ink on an FTO substrate, a black low chalcocite Cu_2_S thin film was obtained, demonstrating an effective conversion from powder into a thin film form (the thin film is discussed in the following section).

**Figure 2:**
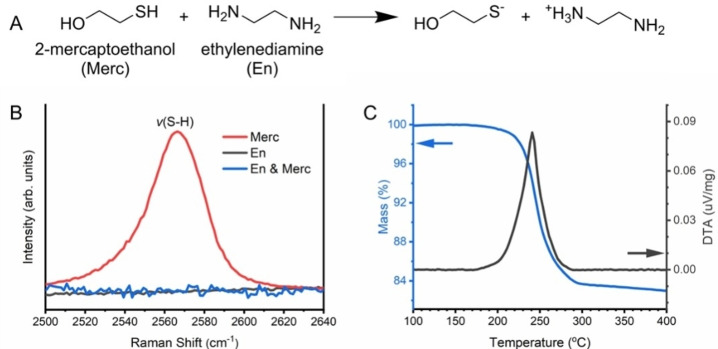
(a) Reaction between 2‐mercaptoethanol (Merc) and ethylenediamine (En), where the amine group deprotonates the thiol group and produces thiolate anions. (b) Molecular structure analysis of Merc, En and En‐Merc mixture by liquid‐phase Raman spectroscopy. (c) TGA and DTA traces of the Cu‐S molecular ink pre‐dried at 160 °C under nitrogen.

It is worth noting that Cu_2_S is insoluble in either Merc or En alone, but soluble in the mixture of Merc and En, indicating that the chemical reaction between Merc and En enabled the dissolution of Cu_2_S. Figure 2A displays the reaction in which the amine group of En deprotonates the thiol group of Merc and produces thiolate anions. The deprotonation of the thiol group is confirmed by liquid‐phase Raman spectroscopy showing that the prominent S−H stretching peak near 2560 cm^−1^ in Merc disappears in the Merc‐En mixture in the Raman spectra in Figure 2B. This observation suggests that the resulting thiolate anions may play an important role in the dissolution of bulk Cu_2_S powder. Regarding the dissolution mechanism of elemental sulfur in a thiol‐amine mixture, it was reported that nucleophilic thiolate opens the eight‐membered sulfur ring to produce alkyl polythiolates (R‐*S_n_* ^−^), followed by the formation of a mixture of alkyl polysulfides (R‐*S_n_*‐R), such as alkyl di‐, tri‐, and tetrasulfides.^[35]^

In addition, these alkyl polysulfides can react with metal cations, such as Sn^2+^, to form molecular clusters containing Sn−S bonds.^[33]^ Thus, it is speculated that the thiolate anions in the Merc‐En mixture break some Cu‐S bonds in Cu_2_S bulk powder by nucleophilic attack, resulting in the formation of molecular Cu‐thiolates, which are soluble in the solvent. Murria et al. identified the molecular structures of copper thiolates upon dissolution of CuCl and CuCl_2_ in a thiol‐amine mixture by mass spectrometry.^[36]^ They proposed various possible Cu‐thiolate molecular clusters, including monometallic and bimetallic clusters. It can be thus assumed that analogous Cu‐thiolate molecules are present in our ink system.

TGA and DTA were further employed to determine the minimum temperature required for the recovery of the solid Cu_2_S phase as well as the possible transformation mechanisms. As shown in Figure 2C, the Cu‐S molecular ink (pre‐dried at 160 °C under nitrogen) displayed a single‐step mass loss that ends around 300 °C, and one obvious exothermic peak appeared in the same region. The temperature of 300 °C is quite similar to that observed in similar thiol‐amine solvent systems for other chalcogenide materials, such as Sb_2_Se_3_, Bi_2_S_3_, and SnS.[^23^, ^25^] In the literature, the strong *ν*(C−H) and *ν*(N−H) stretches at 3300–2800 cm^−1^ disappeared when the inks were heated to 300 °C, indicative of the complete elimination and/or decomposition of the organic species. Thus, the mass loss and exothermic reaction between 160 °C and 300 °C may correspond to the decomposition of the organic species to crystalize Cu_2_S while releasing gaseous products, such as H_2_S, H_2_O and NH_3_.

### Characterization of Cu_2_S thin films

**Figure 3:**
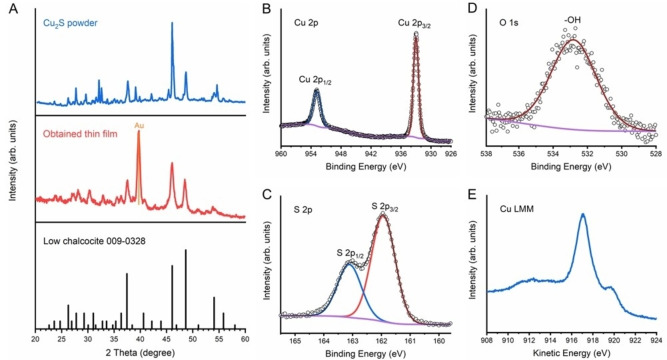
(a) XRD patterns of Cu_2_S powder, the obtained thin film, and low chalcocite JCPDS 009‐0328. (b) Cu2p XPS spectrum. (c) S2p XPS spectrum. (d) O1s XPS spectrum. (e) Cu LMM Auger spectrum.

Figure 3A shows X‐ray diffraction (XRD) patterns of Cu_2_S powder and the obtained thin film by thiol‐amine solution processing. For the powder measurement, we used the highest purity of commercially available Cu_2_S powder and an air‐sensitive sample holder to avoid the oxidation of Cu_2_S (see the experimental section for details). Most of the peaks from Cu_2_S powder, including the three diagnostic peaks at 37.5°, 46°, and 48.5°, are well matched to the low chalcocite Cu_2_S reference (JCPDS 009‐0328), and impurity peaks from CuS and Cu_1.8_S are absent. However, there are also some tiny peaks not being assigned to low chalcocite, such as the peaks at 29.5° and 39°, which correspond to the djurleite Cu_1.96_S phase. It was reported that the stoichiometric low chalcocite phase (Cu_2_S) is thermodynamically unstable in the presence of oxygen and may form copper vacancies, resulting in the formation of djurleite phase (Cu_1.96_S), as Cu atoms near a free surface can react with O_2_ to produce copper oxides.^[37]^ The presence of the djurleite peaks even in high‐purity commercial Cu_2_S powder measured in an air‐sensitive holder indicates the vulnerability of this material to oxidation either during the material synthesis or the XRD measurement. In contrast, in the XRD pattern obtained from the fabricated Cu_2_S thin film measured with grazing incidence mode, only low chalcocite Cu_2_S peaks are observed without any impurity phases, including Cu_1.96_S. One possible explanation for the disappearance of the Cu_1.96_S peaks in the film is its re‐sulfidation owing to the sulfur source (thiolate anions) in the molecular ink during the solution processing. The lower surface area of the obtained thin film compared to that of the Cu_2_S powder during XRD measurements in ambient air might be another reason why Cu_1.96_S is not observed. In any case, the XRD data clearly show that the molecular Cu‐S units in the solution were successfully recovered to the low chalcocite Cu_2_S crystal structure within the thin film form.

To further verify the composition and phase purity of the obtained thin film, X‐ray photoelectron spectroscopy (XPS) and Raman spectroscopy were carried out. XPS of the obtained thin film yielded doublet binding energies of 932.8 and 952.8 eV corresponding to Cu2p_3/2_ and Cu2p_1/2_ (Figure 3B) and doublet peaks at 162.0 and 163.2 eV corresponding to S2p_3/2_ and S2p_1/2_ respectively (Figure 3C), which are consistent with literature values of Cu^+^ and S^2−^ in Cu_2_S.[^4^, ^20^, ^38^] No Cu^2+^ satellites around 965 eV or between 940 and 945 eV were observed in the XPS of Cu2p orbitals. Only one peak at 532.9 eV was observed in the XPS of O1s orbital, which could be associated to hydroxyl groups from surface absorbed water. Consequently, the presence of Cu_2_O can be ruled out due to the absence of a lattice oxygen signal in the O1s core level spectrum.[^38^, ^39^] The Cu LMM Auger spectra also matches well with literature results of Cu_2_S, showing a main peak at a kinetic energy of 916.8 eV as well as a shoulder around 920.0 eV with no visible component at 918.6 eV, which would indicate the presence of metallic Cu.^[38]^ Therefore, the XPS analysis confirms that the obtained thin film is phase pure Cu_2_S without measurable Cu(OH)_2_, CuO, Cu_2_O or Cu impurities.^[38]^ The Raman spectrum of the obtained Cu_2_S thin film showed almost identical peaks to those from Cu_2_S powder at 345, 805 and 1043 cm^−1^ as shown in Figure S1. One clear observation from the Raman spectrum is the absence of S−S stretching at 472 cm^−1^, which is typically observed in the CuS phase. The peaks observed in both the Cu_2_S powder and the obtained thin film might originate from low frequency carbon‐carbon (C−C) vibrations adsorbed on the Cu_2_S surface (i. e. contamination).^[40]^

**Figure 4:**
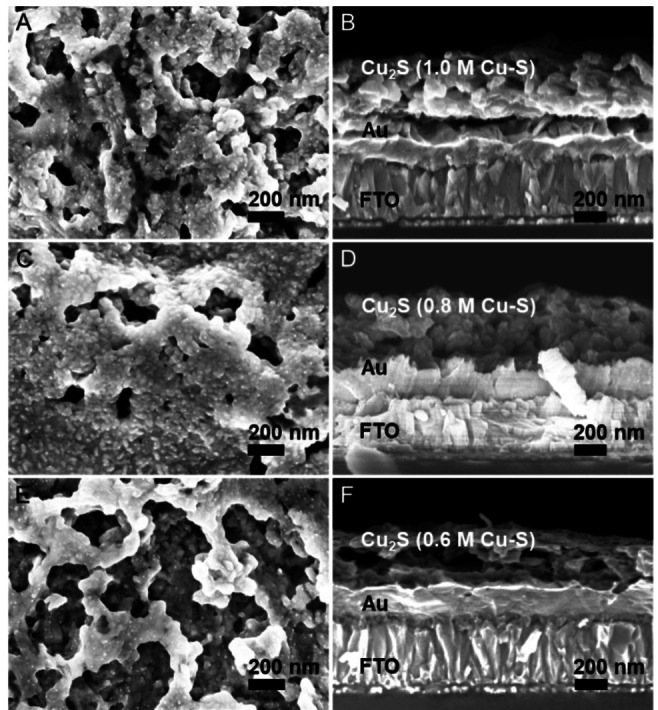
Plan view (a, c, e) and cross‐sectional (b, d, f) SEM images of 3 coats‐Cu_2_S thin films made from 1.0 m (a, b), 0.8 m (c, d) and 0.6 m (e, f) Cu‐S molecular inks.

The optimization of the microstructure and thickness of the light absorber is of critical importance for achieving high‐performance PEC devices due to their influence on light absorption and charge separation. In this regards, three different concentrations of the Cu‐S molecular inks (0.6, 0.8, 1.0 m in Cu‐ion) were prepared as shown in Figure S2. As mentioned above, the Cu‐S molecular inks display a transparent brownish color without any obvious sedimentation. Nevertheless, prior to spin coating on Au substrates, the inks were filtered to remove any undissolved aggregations or impurities. Scanning electron microscopy (SEM) images were taken to check the microstructures and thicknesses of the thin films prepared from the three different concentrations of molecular inks. Figure 4 shows that all thin films exhibit a porous structure composed of nanoparticles with a diameter around 20 nm and a thickness around 400 nm. However, the thin films prepared from the 0.8 and 1.0 m Cu‐S molecular inks appear denser than those prepared from the 0.6 m ink, which is in good agreement with the GIXRD patterns (Figure S3) showing more low chalcocite peaks in higher concentration Cu_2_S thin films.

### Cu_2_S thin film‐based photocathodes

**Figure 5:**
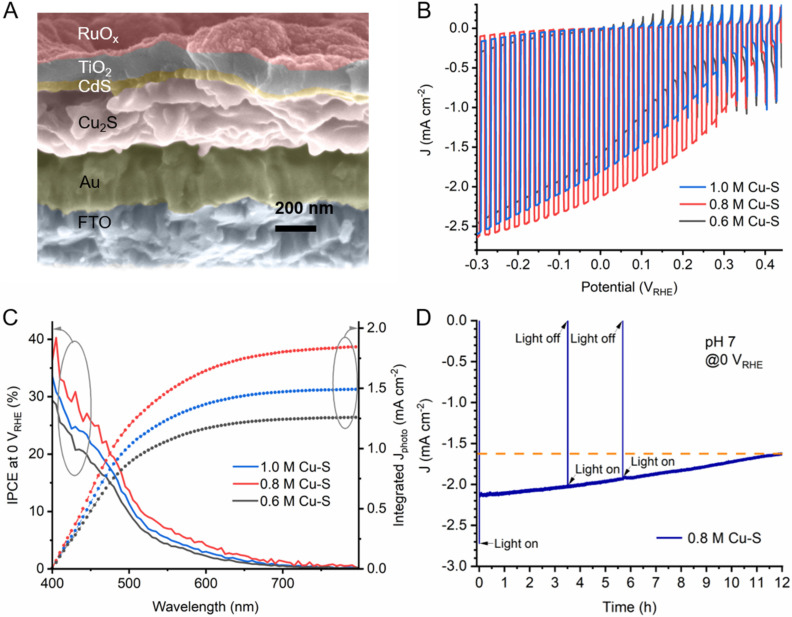
(a) Cross‐sectional SEM image of Cu_2_S photocathode with FTO/Au/Cu_2_S/CdS/TiO_2_/RuO_x_ structure based on Cu_2_S thin film prepared from 0.8 m Cu‐S molecular ink. (b) *J*‐*E* curves of Cu_2_S photocathodes based on Cu_2_S thin films under simulated chopped AM1.5 G illumination (100 mW cm^−2^). (c) Incident photon‐to‐current efficiency spectra of the Cu_2_S photocathodes at an applied bias of 0 V_RHE_ under monochromatic illumination. (d) Measured photocurrent density of the Cu_2_S photocathode based on Cu_2_S thin film prepared from 0.8 m Cu‐S molecular ink under constant bias at 0 V_RHE_ under simulated AM1.5 G illumination (100 mW cm^−2^). All measurements were performed in 1.0 m phosphate buffer solution (pH 7.0).

To prepare Cu_2_S thin film‐based photocathodes, 40 nm of n‐type CdS layer was first deposited on top of the Cu_2_S thin films by chemical bath deposition to form a p‐n junction between Cu_2_S and CdS. Then 100 nm of TiO_2_ was deposited on CdS layer by ALD as a protective layer, and eventually RuO_*x*_ was photoelectrodeposited as the hydrogen evolution catalyst.^[41]^ Figure S4 shows the performance of a bare Cu_2_S photocathode, where it is evident from the low photocurrent and substantial dark current that functional overlayers are needed. Figure 5A shows the cross‐sectional SEM image of the Cu_2_S photocathode based on Cu_2_S thin film prepared from 0.8 m Cu‐S molecular ink, and the cross‐sectional SEM images of Cu_2_S photocathodes based on Cu_2_S thin film prepared from 0.6 and 1.0 m Cu‐S molecular inks are in Figure S5. The thicknesses of Cu_2_S thin films in the photocathodes are around 180, 220, and 300 nm for 0.6, 0.8, and 1.0 m Cu‐S molecular inks, respectively. Regardless of the concentration, all Cu_2_S thin films showed reduced thicknesses after the device fabrication compared to the bare Cu_2_S thin films in Figure 4, probably due to in‐situ etching during the CdS deposition in the presence of NH_3_^.^H_2_O. The different etching rate, evidenced by the different thickness changes, result from the different densities (i. e., porosity) of the Cu_2_S thin films from the different concentrations of ink. Nevertheless, the CdS layer is conformal and compact with a uniform thickness of around 40 nm. The TiO_2_ layer is also homogeneous and was determined to be 100 nm in thickness by ellipsometry on a silicon witness wafer. The RuO_*x*_ catalyst forms a conformal thin film on top of the TiO_2_ layer, which is believed to have better adhesion to the electrode surface and thus enables better stability than the Pt catalyst in the form of nanoparticles.^[41]^

The PEC performance of the Cu_2_S photocathodes were measured in a pH 7 electrolyte (Figure 5). Based on the different thickness of Cu_2_S thin films prepared from 0.6 M, 0.8 m and 1.0 m Cu‐S molecular inks, different photocurrent densities, onset potentials and incident photon to current efficiencies (IPCE) were achieved. The 0.8 m Cu‐S molecular ink‐derived Cu_2_S photocathode performed the best with a photocurrent density of 2.5 mA cm^−2^ at −0.3 V_RHE_ and an onset potential of 0.42 V_RHE_ (see Figure S6 for the detailed determination of the onset potentials), which might be owing to the optimum balance between light absorption and charge transport in the Cu_2_S layer. At the benchmark value of 0 V_RHE_, the photocathode based on Cu_2_S thin film prepared from 0.8 m Cu‐S molecular ink generated a photocurrent density of 2.1 mA cm^−2^, while the values for those based on Cu_2_S thin film prepared from 0.6 and 1.0 m Cu‐S molecular inks were 1.6 and 1.8 mA cm^−2^, respectively. It is likely that 180 nm of Cu_2_S prepared from 0.6 m Cu‐S molecular ink (after in‐situ etching during the CdS deposition) is not thick enough to absorb all of the sunlight, while 300 nm Cu_2_S thin film from the 1.0 m ink is too thick for the photo‐generated carriers to be separated effectively. The minority carrier diffusion length of Cu_2_S is often reported in the range between 100–400 nm,[^42^, ^43^] but values less than 100 nm can also be found in the literature.^[44]^ The short minority diffusion length may result in the decreased performance in thicker Cu_2_S thin film photocathodes.

Figure 5C shows the IPCE spectra and the integrated photocurrent densities of the Cu_2_S photocathodes. All three photocathodes show a response in the visible region with a monotonous decrease as the wavelength increases, as well as a sudden slope change at around 520 nm. The relatively low efficiencies for the photons with wavelength higher than 520 nm are likely due to the deeper photon absorption in the film and insufficient minority carrier diffusion length.^[3]^ It is worth noting that the IPCE values above 700 nm are close to zero. However, as shown in Figure S7, the absorbance above 700 nm remains relatively high while the transmittance is low (<10 %). The discrepancy between the low IPCE and high absorbance near 700 nm indicates that the photons above 700 nm are absorbed by the film but most of the photogenerated electrons and holes recombine due to the short carrier diffusion length. Thus, while a thicker film is desired for more effective photon harvesting, the short minority diffusion length can pose an obstacle for efficient collection of the photo‐generated carriers. The dilemma between light absorption and carrier collection depending on the thickness of Cu_2_S can be resolved by a nanostructuring strategy, which allows superior absorption by light scattering while minimizing the transport distance within the nanostructures. The integrated photocurrent densities calculated from IPCE data of Cu_2_S photocathodes based on Cu_2_S thin films prepared from 0.8 M, 1.0 m and 0.6 m Cu‐S molecular inks are 1.84, 1.49 and 1.26 mA cm^−2^ at 0 V_RHE_, respectively. These values are a bit lower than those in the *J‐E* curves, probably due to the unaccounted‐for photons below 400 nm in the IPCE measurements.

As we mentioned in the introduction section, limited photo‐active Cu_2_S thin films have been reported in the PEC water splitting field so far. The state‐of‐the‐art Cu_2_S thin film‐based photocathodes (FTO/Au/Cu_2_S/CdS/TiO_2_/RuO_*x*_), showing a 7.0 mA cm^−2^ photocurrent at −0.3 V_RHE_ with an onset potential of 0.48 V_RHE_, was reported by Yu et al. using the cation exchange method to prepare the Cu_2_S layer.^[4]^ The higher photocurrent density and onset potential compared with the performance of our Cu_2_S photocathodes could be due to the differences in the preparation methods for either or both of the Cu_2_S and CdS layers. Yu et al. used a modified hydrazine‐based chemical bath method to deposit the CdS layer in order to avoid etching of the Cu_2_S thin films by ammonia, and optimized the thickness of the TiO_2_ layer, resulting in changes in the photocurrent and stability. We believe that the performance of our thiol‐amine based Cu_2_S photocathode could be further enhanced with optimization, but a nanostructuring strategy might be needed to overcome the fundamental limitation of the short carrier diffusion length as we discussed above.

The stability of our best Cu_2_S photocathode was tested with a chronoamperometry measurement at 0 V_RHE_ under simulated AM1.5 G illumination (Figure 5D). After 4 h stability test, the photocurrent density retained 94 %, and after 12 h retained 76 %. The remarkable stability of our Cu_2_S photocathode renders promise for stable solar hydrogen production based on low‐cost Cu_2_S obtained by the ink‐based approach, which is favorable for large area fabrication.

## Conclusion

In summary, we demonstrated that phase‐pure low chalcocite Cu_2_S thin films can be prepared by simple dissolution and recovery processes with thiol‐amine‐based solution processing. After coupling with a CdS buffer layer, TiO_2_ protective layer, and RuO_*x*_ hydrogen evolution catalyst, the champion FTO/Au/Cu_2_S/CdS/TiO_2_/RuO_*x*_ photocathode delivered a photocurrent density of 2.5 mA cm^−2^ at −0.3 V_RHE_, an onset potential of 0.42  V_RHE_, and an extended stability over 12 h under AM1.5 G (100 mW cm^−2^) simulated sunlight illumination. This work provides a simple, cost‐effective, and high‐throughput method to prepare phase‐pure and photoactive Cu_2_S thin films, which will facilitate more studies on Cu_2_S photocathodes for low‐cost and scalable solar‐driven hydrogen fuel.

## Experimental Section

### Preparation of Cu‐S molecular inks

Copper(I) sulfide powder (Cu_2_S, anhydrous, 99.99 %, Sigma‐Aldrich) was dissolved in a solution of 0.4 mL 2‐mercaptoethanol (Merc, 99.0 %, Sigma‐Aldrich) and 1.6 mL 1,2‐ethylenediamine (En, 99.5 %, Sigma‐Aldrich) and stirred overnight at room temperature in a N_2_‐filled glovebox to prepare 0.6, 0.8 and 1.0 m Cu‐S molecular inks (96, 127 and 159 mg of Cu_2_S powder, respectively). The as‐prepared Cu‐S molecular inks were then filtered by 0.2 μm hydrophobic fluorinated (PTFE) filters to remove any undissolved aggregations or impurities.

### Preparation of Cu_2_S thin films

150 nm Au (with 10 nm Cr adhesion layer) was firstly coated on 2.5 cm×2.5 cm FTO as substrates by thermal evaporation at room temperature using an Oxford Vacuum Science VapourPhase/PicoSphere system. The Au/FTO substrates were treated by a UV‐Ozone cleaner for 10 min prior to spin coating. A static dispense spin coating process was applied using a Laurell Technologies Corporation WS‐650Mz‐23NPPB single‐wafer spin processor by uniformly spreading roughly 0.15 mL molecular ink onto the entire Au/FTO substrates and then spin coating at 2000 rpm for 1 min with an acceleration rate of 1200 rpm min^−1^ in a N_2_‐filled glovebox. In between coatings, the films were quickly dried on a hot plate (also in the N_2_‐filled glovebox) at 350 °C for 2 min and then allowed to cool to room temperature before the next coating. When the coating number was fulfilled, a final annealing process at 400 °C for 30 min was performed.

### Chemical bath deposition (CBD) of CdS layer

The homemade setup for the chemical bath deposition of CdS layer consists of a jacketed beaker (1 L internal dimension) and a Thermo Scientific Arctic Series Refrigerated/Heated Bath Circulators (SC150 immersion circulator, A25 stainless steel bath). The Cd solution containing 126.6 mg (0.61 mmol) CdSO_4_, 41.66 mL (1.07 mol) NH_3_ ⋅ H_2_O (ca. 25 %) and 260 mL deionized (DI) water was first prepared in the jacketed beaker and the bath circulator was turned on and stirred at 300 rpm. The bath circulator was allowed to warm up for 30 min until the temperature of the bath reached and remained constant at 60 °C. Then the thiourea solution prepared from 2.85 g (37.4 mmol) thiourea in 33.33 mL DI water was added to the beaker, forming the final CdS deposition aqueous solution of 1.82 mm CdSO_4_, 0.11 m thiourea and 3.19 m NH_3_ ⋅ H_2_O, and allowed to stir for 3 min to get a stable CdS deposition rate. Cu_2_S thin films were then immersed into the solution for another 5 min. After the CdS deposition, the samples were rinsed with DI water, dried under a stream of N_2_, and then immediately put into the ALD reactor for the subsequent deposition of the TiO_2_ layer.

### Atomic layer deposition of TiO_2_ layer

The TiO_2_ layer was deposited by atomic layer deposition (ALD) using a Picosun R200 system. Tetrakis(dimethylamido)titanium (TDMAT) and H_2_O were used as the Ti and O sources, respectively. The temperature of TDMAT precursor cylinder was held at 85 °C, and the reactor temperature was 120 °C. A total of 1860 ALD cycles were carried out to enable the thickness of TiO_2_ layer of roughly 100 nm, which was confirmed by ellipsometry on a silicon witness wafer.

### Photoelectrodeposition of RuO_*x*_ catalyst

For the photodeposition of RuO_*x*_ catalyst, a 1.3 mm potassium perruthenate (KRuO_4_, Alfa Aesar) aqueous solution was used, as described in the literature.^[41]^ The KRuO_4_ solution was freshly prepared before each deposition. Typically, the catalyst was deposited at a constant current density of −28.3 μA cm^−2^ for 15 min under simulated one sun illumination.

### Molecular structure and thermal analysis of Cu‐S molecular inks

The molecular structures of Cu‐S molecular inks were identified by liquid‐phase Raman spectra using a Renishaw System at an exciting wavelength of 532 nm with the laser spot size around 2 μm. Thermal Gravimetric Analysis (TGA) and differential thermal analysis (DTA) were performed on a Netzsch Jupiter STA 449 F3 TGA system with an alumina crucible under a flowing nitrogen atmosphere with a heating rate of 5 °C/min. The sample was pre‐dried at 160 °C under flowing nitrogen prior to TGA/DTA analysis.

### Crystal structure and morphology characterization of Cu_2_S thin films

The crystal structures of Cu_2_S powder and obtained Cu_2_S thin films were examined by X‐ray diffraction using a Rigaku smartlab diffractometer at 2° min^−1^ with a step width of 0.01°, and the Cu_2_S powder was stored in an air‐sensitive sample holder and sealed inside the N_2_‐filled glove box to avoid the possibilities of oxidation during XRD measurements. X‐ray photoelectron spectroscopy (XPS) was performed using a Physical Electronics (PHI) Quantum 2000 X‐ray photoelectron spectrometer featuring monochromatic Al_Kα_ radiation, generated from an electron beam operated at 15 kV and 32.3 W. The energy scale of the instrument was calibrated using a Au reference sample. The analysis was conducted at 1×10^−6^ Pa, with an electron take off angle of 45° and a pass energy of 46.95 eV. Core level binding energies were determined by fitting Voigt profiles (GL80) after Shirley background subtraction. Charge neutralization was used throughout the measurement. The Raman spectra were obtained using a Renishaw System at an exciting wavelength of 532 nm with the laser spot size around 2 μm. The scanning electron microscopy (SEM) images were obtained using a Zeiss Gemini 450. The absorbance and transmittance spectra of the Cu_2_S thin film were measured by UV‐visible spectroscopy (Shimadzu UV 3600 Plus).

### Photoelectrochemical characterization of Cu_2_S photocathodes

PEC performance of the Cu_2_S photocathodes was carried out in a three‐electrode electrochemical cell. A 2‐channel potentiostat (BioLogic SP‐300) was used to control the potential of the working electrode. A Pt wire and an Ag/AgCl electrode (KOSLOW, saturated KCl, +0.197 V *vs*. normal hydrogen electrode (NHE)) were used as counter and reference electrode, respectively. The electrolyte used in all PEC measurements was 1 m phosphate buffer (K_2_HPO_4_/KH_2_PO_4_, pH 7). The light source was a 150 W Xe‐lamp (LOT Oriel) equipped with an AM1.5 G filter, and the intensity (100 mW cm^−2^) was calibrated with a standardized silicon solar cell (PV Measurements, USA). Incident photon‐to‐current efficiency (IPCE) was measured in a home‐built system equipped with a halogen light source and a double monochromator. The light intensity was measured with a calibrated silicon photodiode before each measurement. The stability test of Cu_2_S photocathode made from 0.8 m Cu‐S molecular ink was measured at 0 V_RHE_ in the same setup as that for PEC measurement.

## Conflict of interest

The authors declare no conflict of interest.

## Supporting information

As a service to our authors and readers, this journal provides supporting information supplied by the authors. Such materials are peer reviewed and may be re‐organized for online delivery, but are not copy‐edited or typeset. Technical support issues arising from supporting information (other than missing files) should be addressed to the authors.

Supporting Information
