# Supplementary material for: Thiol‐Amine‐Based Solution Processing of Cu2S Thin Films for Photoelectrochemical Water Splitting
Source: ChemSusChem. 2021 Aug 26;14(18):3967–74. doi: 10.1002/cssc.202101347 (PMC8518488; doi:10.1002/cssc.202101347)
Supplement: Supplementary file 1 — Supporting Information [file CSSC-14-3967-s001.pdf]

ChemSusChem

Supporting Information

Thiol-Amine-Based Solution Processing of Cu_2S Thin Films for Photoelectrochemical Water Splitting

Xi Zhang, Wooseok Yang,* Wenzhe Niu, Pardis Adams, Sebastian Siol, Zhenbin Wang, and S. David Tilley*© 2021 The Authors. ChemSusChem published by Wiley-VCH GmbH. This is an open access article under the terms of the Creative Commons Attribution License, which permits use, distribution and reproduction in any medium, provided the original work is properly cited.

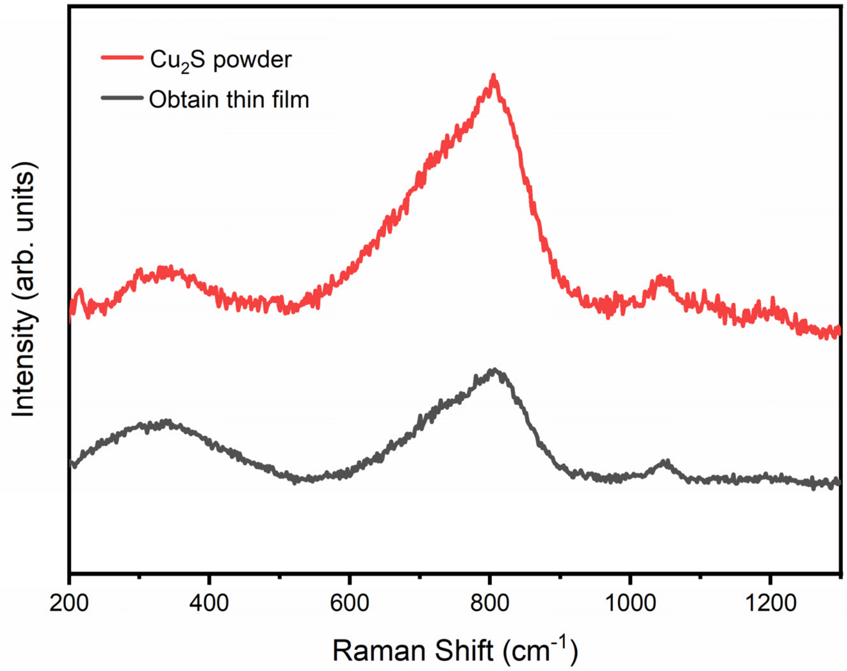

Figure S1. Raman spectra of Cu_2S powder and the obtained thin film.

WILEY-VCH

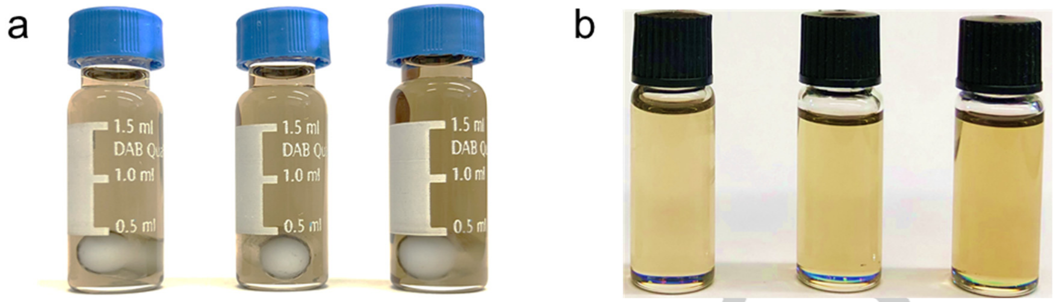

Figure S2. Optical images of 0.6, 0.8 and 1.0 M Cu-S molecular inks (a) before and (b) after filtering with 0.2 μm PTFE filters.

WILEY-VCH

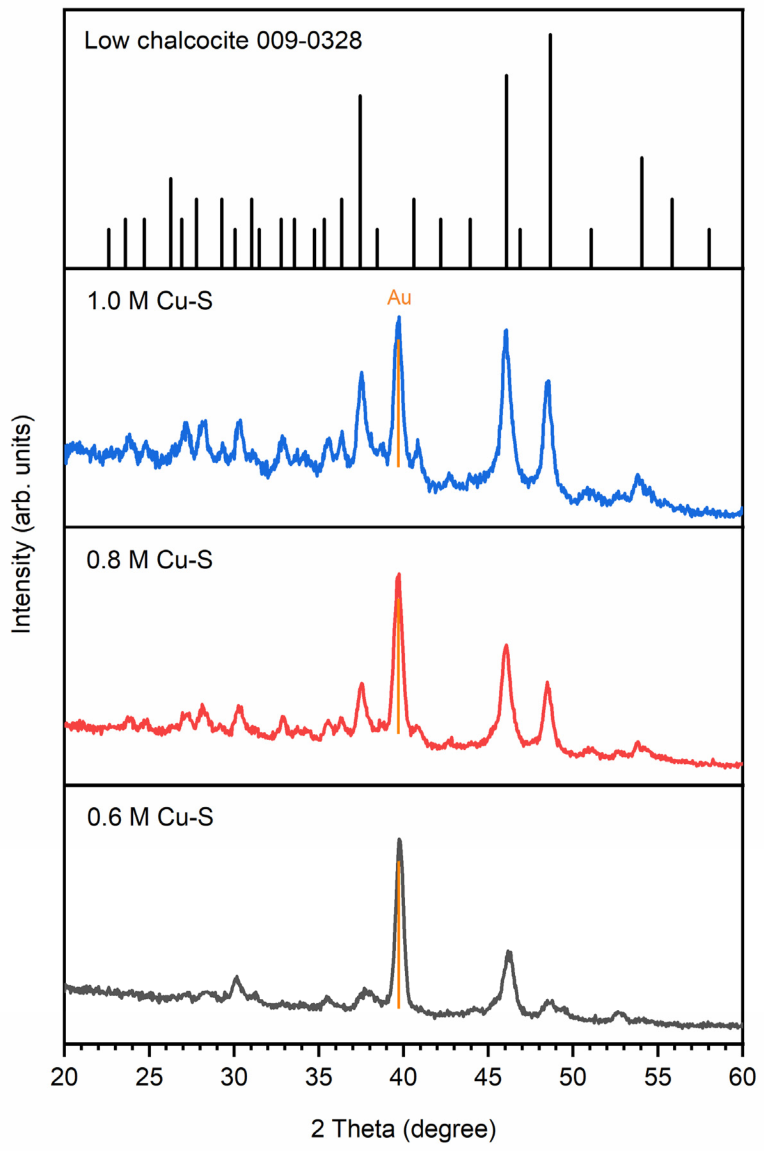

Figure S3. Grazing incidence XRD patterns of 3 coats-Cu_2S thin films prepared from 0.6 M, 0.8 M and 1.0 M Cu-S molecular inks and low chalcocite JCPDS 009-0328.

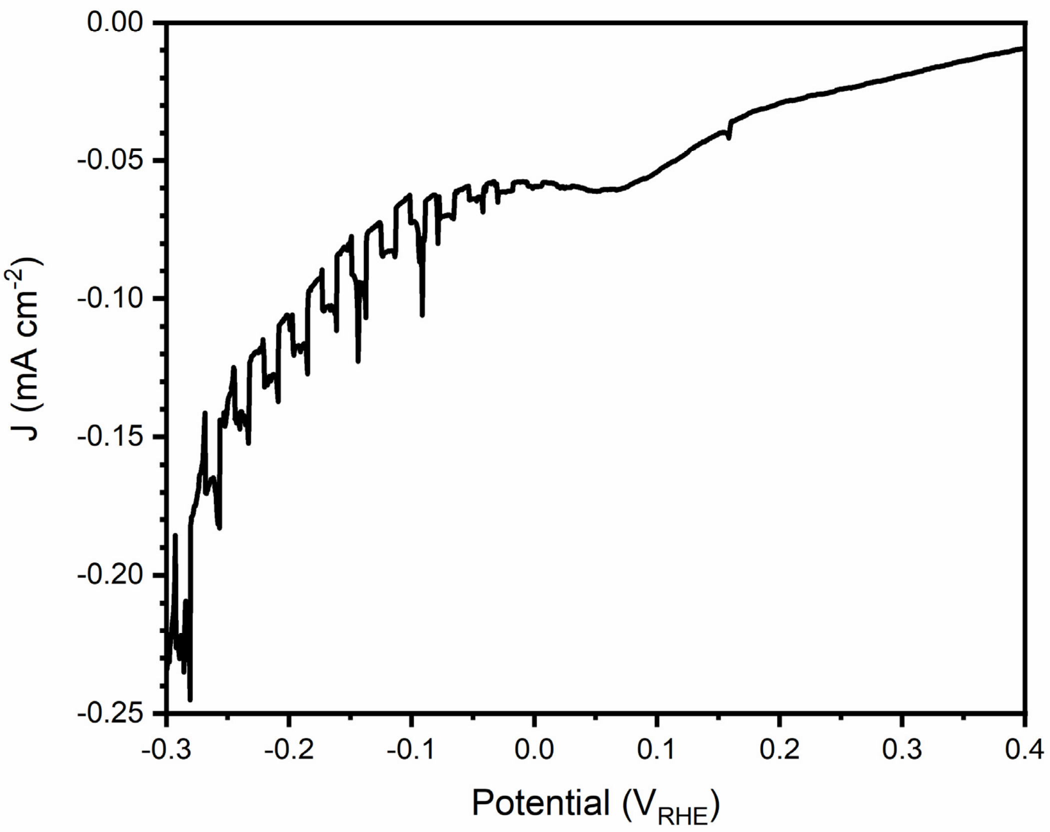

Figure S4. *J*-E curve of the bare Cu_2S thin film prepared from 0.8 M Cu-S molecular ink under simulated chopped AM 1.5 G illumination (100 mW cm^{-2}).

WILEY

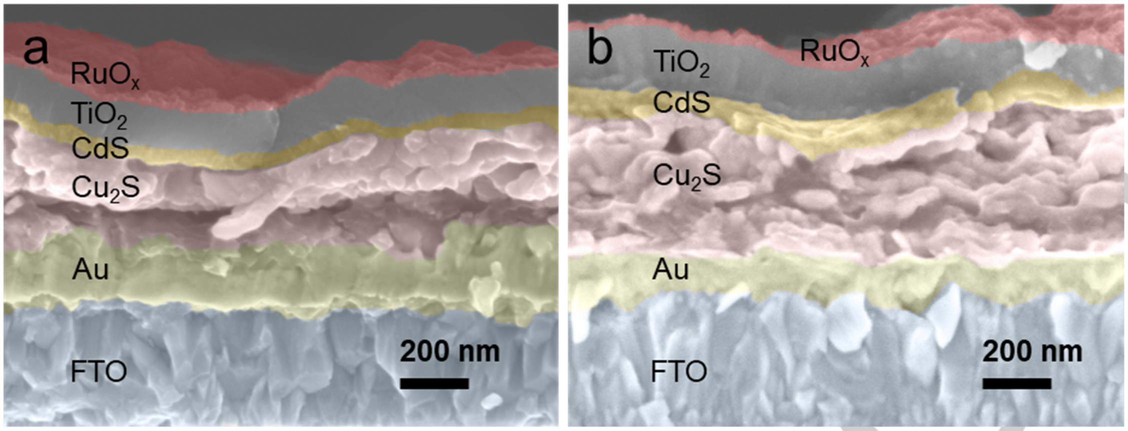

Figure S5. Cross-sectional false-colored SEM images of Cu_2S photocathodes based on Cu_2S thin films prepared from (a) 0.6 M and (b) 1.0 M Cu-S molecular inks.

WILEY-VCH

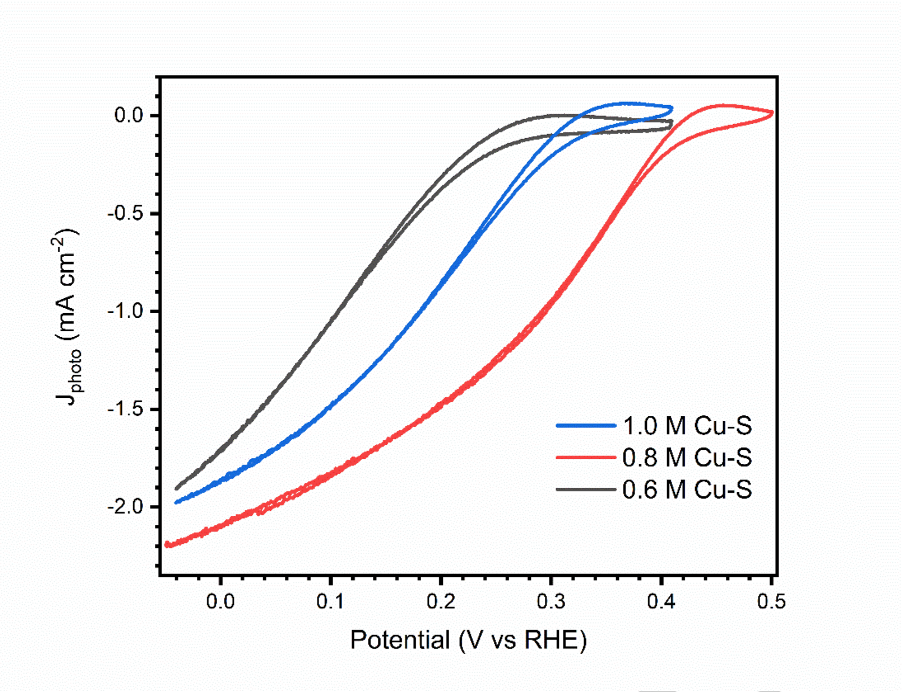

Figure S6. Cyclic voltametry (CV) scans of Cu_2S photocathodes based on Cu_2S thin films prepared from 0.6 M, 0.8 M and 1.0 M Cu-S molecular inks.

WILEY-VCH

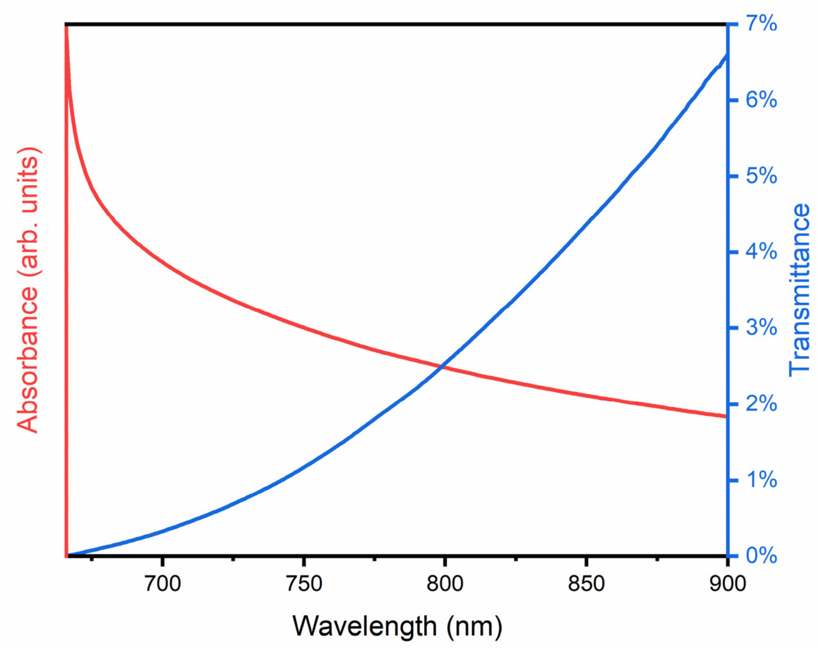

Figure S7. UV-Vis absorbance and transmittance spectra of the Cu_2S thin film prepared from 0.8 M Cu-S molecular ink measured in transmission mode.

WILEY
